# Health practitioners’ perceptions of structural barriers to the identification of intimate partner abuse: a qualitative meta-synthesis

**DOI:** 10.1186/s12913-022-07491-8

**Published:** 2022-01-22

**Authors:** Naomi Hudspeth, Jacqui Cameron, Surriya Baloch, Laura Tarzia, Kelsey Hegarty

**Affiliations:** 1 Department of General Practice, The University of Melbourne, Carlton, VIC Australia; 2 School of Health and Society, University of Wollongong, Wollongong, NSW Australia; 3 Centre for Family Violence Prevention, The Royal Women’s Hospital, Parkville, VIC Australia

**Keywords:** Intimate partner abuse, Intimate partner violence, Health practitioners, Qualitative Meta-synthesis, Barriers

## Abstract

**Background:**

Health care practitioners (HCPs) play a critical role in identifying and responding to intimate partner abuse (IPA). Despite this, studies consistently demonstrate a range of barriers that prevent HCPs from effectively identifying and responding to IPA. These barriers can occur at the individual level or at a broader systems or organisational level. In this article, we report the findings of a meta-synthesis of qualitative studies focused on HCPs’ perceptions of the structural or organisational barriers to IPA identification.

**Methods:**

Seven databases were searched to identify English-language studies published between 2012 and 2020 that used qualitative methods to explore the perspectives of HCPs in relation to structural or organisational barriers to identifying IPA. Two reviewers independently screened the articles. Findings from the included studies were analysed using Thomas and Hardin’s method of using a thematic synthesis and critiqued using the Critical Appraisal Skills Program tool for qualitative studies and the methodological component of the GRADE-CERQual.

**Results:**

Forty-three studies conducted in 22 countries informed the review. Eleven HCP settings were represented. Three themes were developed that described the structural barriers experienced by HCPs: *The environment works against us* (limited time with patients, lack of privacy); *Trying to tackle the problem on my own* (lack of management support and a health system that fails to provide adequate training, policies and response protocols and resources), *Societal beliefs enable us to blame the victim* (normalisation of IPA, only presents in certain types of women, women will lie or are not reliable).

**Conclusion:**

This meta-synthesis highlights the need for structural change to address these barriers. These include changing health systems to enable more time and to improve privacy, training, policies, and referral protocols. On a broader level IPA in health systems is currently not seen as a priority in terms of global burden of disease, mortality and morbidity and community attitudes need to address blaming the victim.

**Supplementary Information:**

The online version contains supplementary material available at 10.1186/s12913-022-07491-8.

## Background

Intimate partner abuse (IPA), also known as intimate partner violence (IPV) is a global public health problem of epidemic proportions, affecting one-third of women worldwide [1]. IPA is characterised as any behaviour by a current or former intimate partner that causes physical, psychological or sexual harm to the other [2]. Although IPA can affect anyone in a relationship, it is a gendered occurrence principally carried out by a man against a female partner [1]. Globally, IPA is widespread in all settings and among all socioeconomic, religious and cultural groups. This prevalence, in combination with the harms is causes to women, families and communities, clearly positions IPA as an urgent issue, requiring a multisectoral response [1].

Exposure to IPA is associated with a range of short and long-term psychological, physical, sexual and reproductive health consequences for women [1]. These adverse health effects lead victims to use healthcare services at an increased rate [3, 4]. Additionally, studies suggest that healthcare providers (HCPs) are often the first professionals trusted with disclosure of abuse [5]. As a result, the vital role of the healthcare system in responding to IPA has been increasingly recognised [4]. However, despite this potential, health services have lagged behind other agencies in addressing IPA, with low identification rates relative to prevalence estimates [4].

Many qualitative studies from a range of health care systems and subspecialties have investigated the barriers HCPs encounter identifying women affected by IPA. This literature was synthesised in a systematic review in 2012 by Sprague et al. [6]. This review of 22 studies found five categories: personal barriers, resource barriers, perceptions and attitudes, fears, and patient-related barriers [6]. Another systematic qualitative review in 2018 by Saletti-Cuesta and colleagues focused on opinions and experiences of HCPs in responding to IPA but was restricted to only primary health care settings [7]. More recently [8], the personal barriers experienced by HCPs were synthesised, highlighting feelings of reluctance and frustration and a sense that the work of responding to IPA was beyond their remit. Specifically, the themes identified were: ‘I can’t interfere’, ‘I don’t have control’ and ‘I won’t take responsibility’. In this review, we have chosen to focus solely on the structural or organisational barriers across health settings, thus updating previous reviews with new data [7, 9–12]. Thus, this review explores the research question: *What do health practitioners perceive as the structural barriers to the identification of intimate partner abuse?*

## Methods^1^

### Search strategy

**Fig. 1:**
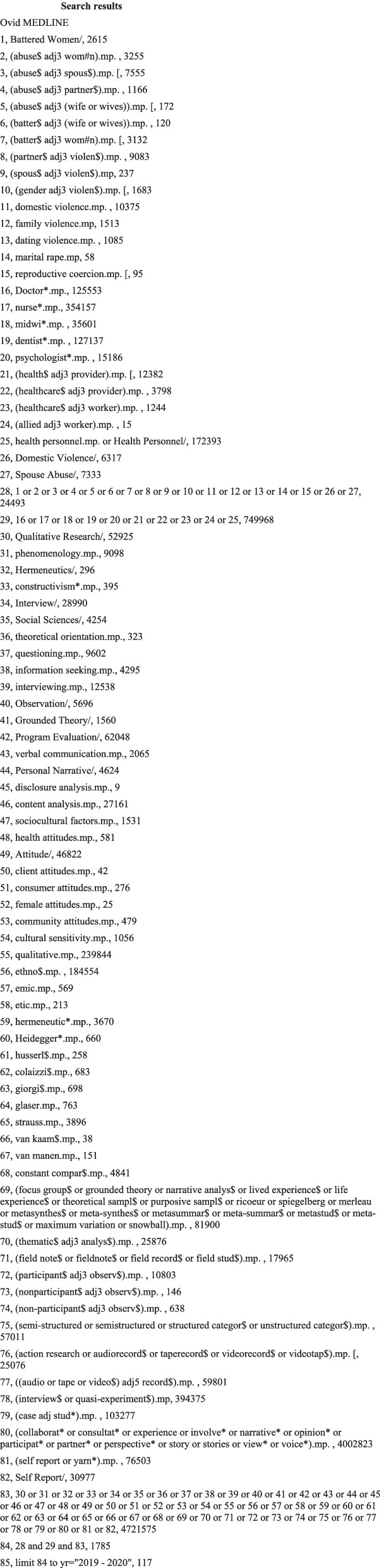
Search results

The protocol for this review was registered with PROSPERO (CRD42020130242). This review’s reporting reflects the Cochrane guidelines for qualitative reviews [13, 14]. The search strategy was informed by our research question. To reflect more contemporary barriers in the health system, a date restriction of 2012–2020 was applied to this review. The search involved three host databases Ovid, EBSCO, and ProQuest (including seven databases). The search comprised of subject headings, text words and keywords for the terms: ‘intimate partner violence/abuse’, ‘qualitative research’, and ‘health practitioners’. No restrictions on geographic location were applied. Studies were included regardless of their publication status, but only English language articles were included. An example of the OVID search is provided in Fig. 1.

### Inclusion criteria

The results generated by the search strategy were imported into the online review management software, Covidence [15], to assist in the management of the large data set. Two reviewers (NH, SB) independently undertook title and abstract screening, followed by a full-text screening applying the following inclusion criteria: (1) a qualitative data collection and analysis method; (2) mixed-methods papers where qualitative data was separate from quantitative data and was qualitatively analysed; (3) survey data with open-ended questions that had been qualitatively analysed; (4) studies of health practitioners (doctors, midwives, allied health workers, nurses, dentists, maternal-child health nurses, Aboriginal health workers, mental health workers); (5) studies that explored instances where a health practitioner is interacting with patients living with intimate partner abuse; (6) studies included findings about barriers for health practitioners addressing intimate partner abuse. Consensus was required for an article to be included in the review. Any disagreements were resolved through discussion with a third reviewer (JC) during the screening process.

### Data extraction and analysis

The data from the primary articles was extracted into a template developed for this review. The extracted information included study setting, sample characteristics, objectives, design, data collection and analysis methods, qualitative themes, qualitative findings, supporting quotations and conclusions. The extraction template was revised on one occasion to accommodate GRADE-CERQual tool details (see Supplementary Material 1).

We began with immersion in the data (reading and examining that data in detail), then subsequently applied the Thomas and Harden [16] thematic synthesis approach; this involved a line-by-line coding of findings from each of the included studies, organisation of initial codes into descriptive codes and generation of analytical themes that involve interpretation to develop further concepts and understandings that answer the research question [16]. After (NH, JC and SB) completed a reading of the included papers in order of publication date, (NH) created initial codes, categories and themes explored by the papers. This data was presented in excel and shared with the wider research team, who met several times (NH, JC, SB, LT, KH) to discuss the development of themes. This process was repeated until consensus was reached. Any disagreements were settled through discussion during the descriptive and analytical coding processes.

### Methodological quality assessment

Three reviewers (NH, SB, JC) independently evaluated the methodological quality of each included study using a modified version of the Critical Appraisal Skills Program (CASP) tool for qualitative studies [17]. Each item was assigned a CASP tool scale; ‘Yes’, ‘Partial’, ‘No’ or ‘Unclear’ designation for eight items related to methodologic quality, and any unaddressed methodologic limitations were named in an open-ended item. The CASP tool was selected because of its capacity to systematically assess the included studies’ validity, the results and their applicability and generalisability to practice [17]. In addition, the level of confidence in the review’s findings was measured through application of the methodological component of the GRADE-CERQual tool [18]. Each included item was categorized as having ‘No or very minor concerns’, ‘Minor concerns’, ‘Moderate concerns’ or ‘Serious concerns.’ Discrepancies in terms of methodological quality were resolved through consultation and discussion with the research team.

## Results

**Fig. 2:**
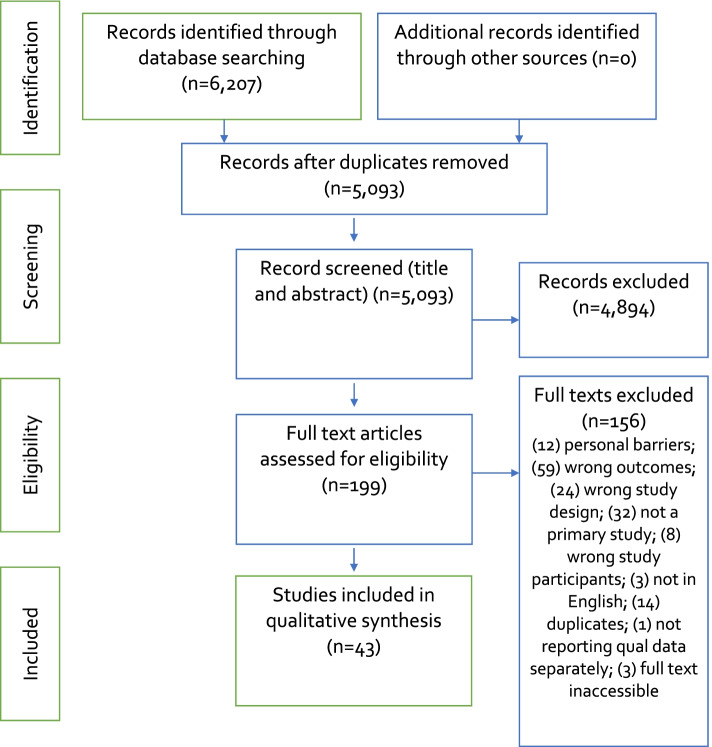
Example of OVID search

We identified 43 studies published between 2012 and 2020. Fig. 2 depicts the search strategy results presented in accordance with the PRISMA (Preferred Reporting Items for Systematic reviews and Meta-Analyses) guidelines [19].

The synthesis included 43 studies conducted in 22 countries. Eleven were conducted in the USA [20–30], four in Canada [31–34], four in the UK [35–38], three in Sweden [39–41], two in Brazil [42, 43], Spain [44, 45] and South Africa [46, 47] respectively. The rest were from Australia [48], China [49], Columbia [50], Egypt [51], Greece [52], Italy [53], Jamaica [54], Lebanon [55], Malaysia [56], Norway [57], Slovenia [58], Sri Lanka [59], The Netherlands [60], Turkey [61] and Zimbabwe [62]. The synthesis included studies with a range of qualitative data collection techniques, including semi-structured interviews, focus group discussions, in-depth interviews, semi-structured focus group discussions, semi-structured telephone interviews and open-ended surveys.

The studies included data from 1563 practitioners with between 1.1 months and 45 years of professional experience across specialisations including emergency medicine, primary care, obstetrics and gynaecology, maternal and child health, family planning, prenatal and antenatal medicine, intensive care, mental health, orthopaedics, and allied health. A summary of the characteristics of the included studies is provided in Table 1.

**Table 1:** Study characteristics

Author	Year (Country)	Study objective	Qualitative Method	Sample	Sample	Years’ experience (mean years)
Arboit et al. [42]	2020 (Brazil)	Determine the potential and limitations of Primary Health Care professionals to identify situations of violence against women.	Semi-structured interview	Health providers/professionals	*n* = 21	< 5
Aziz et al. [51]	2019 (Egypt)	Assess perceptions and practices of screening for DV and to identify predictors of their attitude and behaviour of screening for DV in Assiut University Hospital.	Focus-group discussions	Physicians and nurses	*n* = 22	< 5
Danitz et al. [23]	2019 (USA)	Focus on feedback from a wide array of providers regarding the acceptability and feasibility of RISE, and associated recommendations for refinements of content and context in order to increase the likelihood of the usefulness, acceptability, and feasibility of the RISE intervention to VHA providers, the end-users, should RISE prove to be effective.	Semi-structured telephone interviews	Health providers/professionals	*n* = 2	< 10
Gomez-Fernandez et al. [45]	2019 (Spain)	Use reflections of primary care midwives to know the barriers and facilitators for detecting IPV during pregnancy.	Semi-structured individual interviews	Midwives	*n* = 12	10+
Hatcher et al. [46]	2019 (South Africa)	Explore the views of patients, health providers, and community members around assessing and addressing IPV in this setting.	In-depth interviews and focus group discussions	Health providers/professionals	*n* = 8	N/S
Rahmqvist et al. [40]	2019 (Sweden)	Describe nurses’ experiences when caring for victims of violence and their family members in the emergency department.	Semi-structured interviews	Registered nurses	*n* = 12	10+
Sun et al. [49]	2019 (China)	Investigate the barriers of Chinese PCPs toward managing DV, including barriers of recognition, management, and referrals of these patients.	Focus-group discussions	Primary Care Physicians	*n* = 26	10+
van der Wath [47]	2019 (South Africa)	Uncover discourses that may help understand emergency nurses’ responses towards women exposed to intimate partner violence.	Semi-structured focus group discussions	Nurses	*n* = 15	N/S
Wild et al. [48]	2019 (Australia)	Investigate the barriers midwives face in identifying, enquiring, responding and referring.	In-depth interviews and group discussions	Midwives	*n* = 36	10+
Wyatt et al. [30]	2019 (USA)	Identify if recently licensed registered nurses screen for intimate partner violence, how they screen, which patients are screened, and how pre- licensure education and current workplace training has influenced these screening decisions and behaviours.	Interviews	Nurses	*n* = 16	< 5
Alvarez et al. [21]	2018 (USA)	Describe how healthcare workers serving primarily low-income Latina populations are currently screening and responding to IPV disclosure.	Semi-structured interviews	Health providers/professionals	*n* = 17	10+
Horwood et al. [36]	2018 (UK)	Explore the perceptions and experiences of sexual health clinic staff and DVA advocates after participation in the IRIS ADViSE pilot and to investigate factors which may influence implementation and outcomes.	Semi-structured interviews	Health providers/professionals	*n* = 17	N/S
Henriksen et al. [57]	2017 (Norway)	Gain an in-depth understanding of midwives’ experiences with routine enquiry for intimate partner violence during the antenatal period.	Semi-structured interviews	Midwives	*n* =	< 10
Jack et al. [33]	2017 (Canada)	Develop strategies for the identification and assessment of intimate partner violence in a nurse home visitation programme.	Focus-group and individual interviews	Nurses	*n* = 32	N/S
McCauley et al. [37]	2017 (UK)	Investigate the knowledge and perceptions of domestic violence among doctors who provide routine antenatal and postnatal care at healthcare facilities in Pakistan. In addition, we explored possible management options, enabling factors of and barriers to routine screening of domestic violence.	Semi-structured interviews	Doctors (providing routine antenatal and postnatal care)	*n* = 25	10+
Sundborg et al. [41]	2017 (Sweden)	Improve the understanding of DNs’ experiences of encountering women exposed to IPV.	In-depth interviews	District nurses	*n* = 11	N/S
Williams et al. [28]	2017 (USA)	Examine variations in the implementation of IPV screening practices and to explore both barriers and facilitators that providers experience.	Semi-structured, in-depth interviews	Health providers/professionals	*n* = 10	N/S
Zijilstra et al. [60]	2017 (The Netherlands)	Explore if similar barriers to identification and management of IPV played a role at a Dutch ED to find possible angles for improving care for victims of IPV.	Semi-structured interviews	Emergency Department	*n* = 18	> 5
Al-Natour et al. [20]	2016 (USA)	Describe Jordanian nurses’ roles and practices in screening for intimate partner violence.	Semi-structured interviews	Nurses	*n* = 12	N/S
Bender [22]	2016 (USA)	Explore and describe healthcare providers’ and survivors’ perspectives on IPV with the aim of improving healthcare delivery in rural communities.	Semi-structured interviews	Health providers/professionals	*n* = 7	10+
Fay-Hillier et al. [24]	2016 (USA)	Explore IPV screening practices of RNs who currently work in the ED and what influenced their screening practices.	Semi-structured interviews	Registered Nurses	*n* = 21	10+
Kopcavar et al. [58]	2016 (Slovenia)	Obtain a deeper insight into the attitudes of physicians towards screening for domestic violence. We wanted to identify the barriers to screening for violence of family doctors in their respective populations, and to learn about their experiences and obstacles in the active detection of violence.	Semi-structured interviews	Family doctors (working in rural or urban environments)	*n* = 10	N/S
Pitter [54]	2016 (Jamaica)	Assess midwives’ knowledge and attitudes when encountering GBV in their practice in Kingston, Jamaica.	Focus-group discussions	Midwives	*n* = 6	> 5
Wilson et al. [29]	2016 (USA)	Explore the experiences of healthcare providers who have screened for and/ or addressed IPV with MSFW women patients.	In-depth interviews	Health providers/professionals	*n* = 9	N/S
Visentin et al. [43]	2015 (Brazil)	Identify the actions conducted by primary health care nurses for women in situations of domestic violence.	Semi-structured interviews	Nurses	*n* = 17	> 10
Briones-Vozmediano et al. [44]	2014 (Spain)	Explore the experience of service providers in Spain regarding their daily professional encounters with battered immigrant women as well as their perception of this group’s help-seeking process and the eventual abandonment of the same.	In-depth interviews and focus-group discussions	Health providers/professionals	*n* = 9	N/S
Gotlib Conn et al. [32]	2014 (Canada)	Identify knowledge gaps, perceived barriers and enablers for practising IPV screening in the clinical orthopaedic setting.	Focus-group discussions	Orthopedic surgeons	*n* = 18	10+
Mauri et al. [53]	2015 (Italy)	Explore midwives’ knowledge and clinical experience of domestic violence among pregnant women, with particular emphasis on their perceptions of their professional role.	Semi-structured interviews	Midwives	*n* = 15	10+
McCall-Hosenfeld et al. [26]	2014 (USA)	Assess the opinions and practices of primary care physicians caring for rural women with regard to IPV identification, the scope and severity of IPV as a health problem, how PCPs respond to IPV in their practices, and barriers to optimized IPV care in their communities.	Semi-structured interviews	Internists, family practitioners and obstetrician-gynecologists	*n* = 19	10+
Papadakaki et al. [52]	2014 (Greece)	Explore the perceptions and practices of general practitioners (GPs) regarding the identification and management of victimized patients in primary care settings.	Focus-group interviews	General Practitioners	*n* = 18	10+
Ramachandran et al. [27]	2013 (USA)	Describe screening practices and factors that influence this process among health care workers in sexual and reproductive health clinics in Baltimore City.	In-depth interviews	Healthcare providers (nurses)	*n* = 14	N/S
Usta et al. [55]	2014 (Lebanon)	Explore physicians’ attitudes about responding to DV, their perception of the physician’s role, and the factors that influence their response.	Semi-structured interviews	Health providers/professionals	*n* = 67	> 5
Baird et al. [35]	2013 (UK)	Evaluate the degree to which practice changes identified in the 2004/ 2005 evaluation of the Bristol Pregnancy Domestic Violence Programme (BPDVP) for routine enquiry for domestic abuse have been maintained.	Focus-group interviews	Midwives	*n* = 11	10+
Colombini et al. [56]	2013 (Malaysia)	Explore the views and attitudes of health providers towards IPV and abused women, and to investigate their impact on the provision and the quality of OSCC integrated services in Malaysia.	In-depth interviews	Health providers/professionals	*n* = 54	N/S
Iverson et al. [25]	2013 (USA)	Provides an initial qualitative assessment of VHA primary care providers’ perspectives regarding IPV screening practices.	In-depth semi-structured interviews	Health providers/professionals	*n* = 1	10+
Shamu et al. [62]	2013 (Zimbabwe)	Explore perceptions and experiences of nurse midwives working in Zimbabwe’s public maternity services regarding IPV among pregnant women, including possible responses in the clinic setting.	In-depth interviews, focus-group discussion and observation	Midwives	*n* = 6	N/S
Sprague et al. [34]	2013 (Canada)	Explore perceived barriers to IPV screening in the orthopaedic fracture clinic and by identifying potential facilitators for addressing these barriers among orthopaedic surgeons and surgical trainees (senior and junior orthopaedic residents).	Focus-group discussions	Orthopedic surgeons and strainees (senior and junior orthopedic residents).	*n* = 20 (mean 10)	10+
Beynon et al. [31]	2012 (Canada)	Explore physicians’ and nurses’ experiences, both professional and personal, when asking about IPV; determine the variations by discipline; and identify implications for practice, workplace policy and curriculum development.	Open ended survey	Physicians and nurses	*n* = 769	N/S
Efe-Taskin et al. [61]	2012 (Turkey)	Delineate the factors that prevent the adequate provision of nursing services to women subjected to domestic violence.	In-depth interviews	Nurses	*n* = 30	< 5
Finnbogadottir et al. [39]	2012 (Sweden)	Explore midwives’ awareness of and clinical experience regarding domestic violence among pregnant women in southern Sweden.	Focus-group discussions	Midwives	*n* = 16	10+
Guruge [59]	2012 (Sri Lanka)	Explore the research questions: (1) What are nurses’ perceptions of their role in caring for women experiencing IPV in the Sri Lankan context; (2) What are the barriers nurses face in providing appropriate care to women living with IPV in the Sri Lankan context?	Open-ended, unstructured interviews	Nurses	*n* = 30	10+
Yeung et al. [38]	2012 (UK)	Explore the perceptions and experiences of general practitioners (GPs) and practice nurses on addressing domestic violence before and after participation in a domestic violence training and support programme.	Semi-structured interviews	Health providers/professionals	*n* = 17	> 5

### Quality of included studies

Individual study quality was assessed using a modified Critical Appraisal Skills Programme (CASP) checklist for qualitative studies [17] and the methodological component of the GRADE-CERQual tool [18]. Each theme was appraised including thirty-two studies that had ‘no or very minor concerns’, ten studies were appraised as having ‘minor concerns’, one study was appraised as having ‘moderate concerns’ and no studies were appraised as having ‘serious concerns’. The minor concerns stemmed from ethical considerations for example, recruitment strategies and linkages between researcher and participants. However, all the studies included a clear statement of the aims, had qualitative methodology and research design that was appropriate to address the aims of the research. Please see Table 2 for the combined CASP and GRADE-CERQual results.

**Table 2:** CASP & GRADE-CERQual

Author	Statement of aim	Qualitative methodology appropriate	Research design appropriate	Recruitment strategy appropriate	Relationship between researcher & participants adequately considered	Ethical issues taken into consideration	Data analysis sufficiently rigorous	Findings supported by evidence	Other limitations	GRADE-CERQual assessment of quality
Al-Natour et al.	Yes	Yes	Yes	Yes	Yes	Yes	Yes	Yes	Limited data supporting themes	No or very minor concerns
Alvarez et al.	Yes	Yes	Yes	Yes	Yes	Yes	Yes	Yes	N/A	No or very minor concerns
Arboit et al.	Yes	Yes	Yes	Unclear	Yes	Yes	Yes	Yes	Limited details on recruitment	No or very minor concerns
Aziz & El-Gazzar	Yes	Yes	Yes	Yes	Yes	Yes	Yes	Yes	Low generalisability	No or very minor concerns
Baig et al.	Yes	Yes	Yes	Yes	Partial	Yes	Yes	Yes	N/A	No or very minor concerns
Baird et al.	Yes	Yes	Yes	Yes	Yes	Yes	Partial	Yes	N/A	No or very minor concerns
Bender	Yes	Yes	Yes	Yes	Yes	Yes	Yes	Yes	N/A	No or very minor concerns
Benyon et al.	Yes	Yes	Yes	Yes	Yes	Yes	Yes	Yes	N/A	No or very minor concerns
Briones-Vozmediano et al.	Yes	Yes	Yes	Yes	Yes	Yes	Yes	Yes	N/A	No or very minor concerns
Columbini et al.	Yes	Yes	Yes	Unclear	Yes	Yes	Yes	Yes	N/A	No or very minor concerns
Danitz et al.	Yes	Yes	Yes	Yes	Yes	Yes	Yes	Yes	N/A	No or very minor concerns
Efe & Taskin	Yes	Yes	Yes	yes	Unclear	Partial	Yes	Yes	No details on ethics and data analysis	Minor concerns.
Fay-Hillier et al.	Yes	Yes	Yes	Yes	Yes	Yes	Yes	Yes	N/A	No or very minor concerns
Finnbogadottir & Dykes	Yes	Yes	Yes	Yes	Yes	Yes	Yes	Yes	N/A	No or very minor concerns
Gomez-Fernandez et al.	Yes	Yes	Yes	Unclear	Unclear	Yes	Yes	Yes	Recruitment not clear	Minor concerns.
Gotlib Conn et al	Yes	Yes	Yes	Yes	Yes	Yes	Yes	Yes	N/A	No or very minor concerns
Guruge	Yes	Yes	Yes	Yes	Yes	Yes	Yes	Yes	N/A	No or very minor concerns
Hatcher et al.	Yes	Yes	Yes	Yes	Unclear	Yes	Yes	Yes	Potential risk of bias	No or very minor concerns
Henriksen et al.	Yes	Yes	Yes	Yes	Yes	Yes	Yes	Yes	N/A	No or very minor concerns
Horwood et al.	Yes	Yes	Yes	Yes	Yes	Yes	Yes	Yes	N/A	No or very minor concerns
Iverson et al.	Yes	Yes	Yes	Yes	Yes	Yes	Yes	Yes	N/A	No or very minor concerns
Jack et al.	Yes	Yes	Yes	Yes	Yes	Yes	Yes	Yes	N/A	No or very minor concerns
Kopcavar et al.	Yes	Yes	Yes	Yes	Yes	Yes	Yes	Yes	N/A	No or very minor concerns
Mauri et al.	Yes	Yes	Yes	Yes	Partial	Yes	Yes	Yes	No limitations acknowledged	No or very minor concerns
McCall-Hosenfeld et al.	Yes	Yes	Yes	Yes	Yes	Yes	Yes	Yes	N/A	No or very minor concerns
McCauley et al.	Yes	Yes	Yes	Yes	Yes	Yes	Yes	Yes	N/A	No or very minor concerns
Papadakaki et al.	Yes	Yes	Partial	Yes	Yes	Yes	Yes	Yes	There was no means to control for bias	Minor concerns
Pitter	Yes	yes	Yes	Partial	yes	Yes	Partial	Partial	Concerns around sample recruitment, data analysis and discussion	Moderate concerns
Rahmqvist et al.	Yes	yes	yes	yes	yes	yes	yes	yes	N/A	No or very minor concerns
Ramachandran et al.	Yes	Yes	Yes	Yes	Unclear	Unclear	yes	Yes	N/A	Minor concerns
Shamu et al.	Yes	Yes	Yes	Yes	Unclear	Yes	Yes	Yes	Limitations not clear	Minor concerns
Sprague et al.	Yes	Yes	Yes	Yes	Yes	Yes	Yes	Yes	N/A	No or very minor concerns
Sun et al.	Yes	Yes	Yes	Yes	Partial	Partial	Partial	Yes	Concern around ethical issue and data collection	Minor concerns
Sundborg et al.	Yes	Yes	Yes	Yes	Yes	Yes	Yes	Yes	N/A	No or very minor concerns
Usta et al.	Yes	Yes	Yes	Yes	Partial	Yes	Yes	Yes	Concerns about data collection	Minor concerns
Van der Wath	Yes	Yes	Yes	Partial	Partial	Yes	Yes	Yes	N/A	No or minor concerns
Visentin et al.	Yes	Yes	Yes	Yes	Yes	Yes	Yes	Yes	N/A	No or very minor concerns
Wild et al.	Yes	Yes	Yes	Yes	Unclear	Yes	Yes	Yes	Potential risk of bias	No or very minor concerns
Williams et al.	Yes	Yes	Yes	Yes	Yes	Yes	Partial	Yes	Limited quotes to support evidence	Minor concerns
Wilson et al.	Yes	Yes	Yes	Yes	Yes	Yes	Yes	Yes	N/A	No or very minor concerns
Wyatt et al.	Yes	Yes	Yes	Yes	Unclear	Yes	Yes	Yes	Potential risk of bias	No or very minor concerns
Yeung et al.	Yes	Yes	Yes	Unclear	Yes	Unclear	Yes	Yes	N/A	Minor concerns
Zijlstra et al.	Yes	Yes	Yes	Yes	Yes	Partial	Yes	Yes	Concerns about ethical approval	Minor concerns

### Key themes

Thematic synthesis of the included studies led to the development of three key themes that describe the structural barriers identified by HCPs as preventing them from responding to IPA. These are: *The environment works against us* (limited time with patients, lack of privacy); *Trying to tackle the problem on my own* (lack of management support and a health system that fails to provide adequate training, policies and response protocols and resources), *Societal beliefs enable us to blame the victim* (normalisation of IPA, only presents in certain types of women, women will lie or are not reliable). A table of themes and subthemes is provided in Table 3.

**Table 3:** Summary of themes & subthemes

Theme	Studies contributing
*The environment works against us* - Limited time with patients - Lack of privacy	43 papers [21–24, 26–32, 34–40, 42–50, 52–62]
*Trying to tackle the problem on my own* - Lack of management support - Health system failure to provide adequate training, polices and response protocols and resources	36 papers [20, 21, 23, 25–28, 30–36, 38–41, 43–49, 51–54, 56, 57, 59–63]
*Societal beliefs enable us to blame the victim* - Normalisation of IPA - Belief that IPA only present in certain types of women - Women will lie or are not reliable	20 papers [22, 24–26, 29, 30, 37, 38, 40–42, 44, 47, 53, 54, 57–60, 62]

#### The environment works against us

This theme focuses on the issues experienced ‘on the ground’ by HCPs. It was the largest theme identified in 38 papers of the 43 studies examined [21–24, 26–32, 34–40, 42–50, 52–62]. Consistent with previous reviews, our synthesis highlighted several structural barriers at the level of the healthcare environment that impacted on HCPs’ interactions with patients.

HCPs across most healthcare settings highlighted time constraints as a major problem impeding IPA identification and response. Many participants lamented short clinic appointment times, increased workloads, and the nature of limited patient interactions, highlighting that these prevented the establishment of rapport. Although time barriers were emphasised more amongst HCPs working in settings such as the emergency department, even primary care physicians (e.g. general practitioners/family doctors) and nurses raised it as an issue.

*Doctors who only have ten minutes to spend with their patients—they can’t ask about intimate partner violence. Even if they did, nobody would open up to them about a personal matter like that in ten minutes* [22]*. (Nurse, USA)*

*It’s hard to develop a feeling of trust in a short period of time* [34]*. (Orthopedic surgeon/trainee, Canada)*

*I have more than enough to do without digging too deep. The topic is big and difficult. It is big and difficult and takes time, right?... If somebody discloses things you need to make time to address it* [57]*. (Midwife, Norway)*

For some HCPs, the lack of time was such a problem that it was preferable to discourage disclosures rather than be forced into a position where they could not address them properly. A nurse working in the sexual health setting in the UK commented that:

*There are ways to ask the question to get a negative answer if you’re in a hurry* [36]*. (Sexual health nurse, UK)*

HCPs across multiple healthcare settings highlighted lack of privacy as another critical barrier to IPA identification. HCPs pointed out that women often attended appointments with their partner, which made it inappropriate and potentially unsafe to ask about IPA.

*Sometimes…I’ll ask [about IPA], just because it’s a legality issue, but a lot of times—for instance, if you’re married and you come to the ER, chances are you and your husband are both coming in the triage room. So [if I] say, “Are you a victim of domestic violence or abuse?” you’re probably not going to answer at that time honestly, if you are* [24]*. (Emergency department nurse, USA)*

*…Sometimes the husband is there too, which makes one wonder what is going to happen to the woman afterwards, will it become worse if I dig into this right now?* [39]*. (Midwife, Sweden)*

Even when women attended alone, the physical environment within many healthcare settings was itself a barrier to sensitive inquiry. Poor design, noise, and constant interruptions made it difficult for HCPs to have sensitive discussions with women about IPA. A midwife working in the Spanish sexual and reproductive healthcare setting, for example, noted that in her clinic:

*There are 3 doors, plus a telephone that rings all of the time, [but] when a woman is describing a situation like this, then nothing should interrupt her visit* [45]*. (Midwife, Spain)*

Similarly, a study exploring the perspectives of orthopaedic surgeons and trainees in the US fracture clinic setting, described a clear example of these issues:

*“There’s six other people, at least six plus learners so probably twelve people listening to every single conversation I have with patients; it’s not the appropriate place”. In addition, many fracture clinics follow an open concept model, with curtains separating exam rooms. One participant made the following analogy: “The fracture clinic is the equivalent of a family doctor seeing patients in their waiting office* [34]*. (Surgeons, Canada)*

In the rural context, HCPs also suggested that a lack of confidentiality was a barrier to IPA identification. They pointed out that because “everybody knows everybody” [22] in a small community, that women experiencing IPA may be reluctant to disclose to a HCP they know socially or to have information recorded on their chart.

#### Trying to tackle the problem on my own

This theme, reflected in 36 of the included studies [12, 20, 21, 23, 25–28, 30–36, 38–41, 43–49, 51–54, 56, 57, 59–62], highlights that feeling unsupported by colleagues, the organisation and the health system more broadly was a key barrier to identifying IPA.

Participants across 35 of the studies felt that they were ill-prepared to tackle the challenging work of IPA identification and response, and perceived that the health sector did not prioritise education and training highly enough [12, 20, 21, 23, 25–28, 30–36, 38, 39, 41, 43–49, 51–54, 56, 57, 59–62]. This neglect began early when HCPs were still studying. Practitioners emphasised that the majority of their professional training had provided limited or no content in responding to IPA [49].

*I think the biggest thing is it's really not touched on a lot in school* [30]*. (Nurse, Australia)*

*I think that our education about domestic violence is somehow...lacking* [53]*. (Midwife, Italy)*

Further, many HCPs felt that their workplaces and organisations also did not prioritise ongoing education. Participants in a variety of settings and in different countries expressed a desire to receive additional training to improve their confidence in identifying and responding to IPA but suggested that this was neither offered nor encouraged. For example, an emergency department nurse in a Turkish study suggested that:

*You need to be trained for this. I don’t know, something like a course...I haven’t done anything at the moment so I don’t know how adequate I would be* [61]*. (Nurse, Turkey)*

Similarly, a midwife in a Norwegian study by Henriksen and colleagues [57] expressed frustration that she was being asked to screen patients without being provided with any support or training.

*I feel that this is something we just have to deal with without anyone telling us how to do it. So I think that I feel provoked that they [the organization] have just decided this without training us properly* [57]*. (Midwife, Norway)*

In addition to a lack of training opportunities, HCPs lamented the absence of comprehensive IPA policies and protocols to guide practitioners in identification and response. This led to feelings of uncertainty and confusion. One practitioner from a study by Rahmqvist and colleagues [40] in the Swedish emergency department setting commented that:

*I would like to know exactly what to do, with clear routines so that when it comes up, that they have been victimized, I know what to do. How can I help? Where can I refer the patient for follow up care?... sometimes it hasn’t worked out before, so I hesitate to ask or engage because I don’t know what to do or what will happen if I try to refer* [40]*. (Emergency department nurse, Sweden)*

A sexual health nurse in an American study by Ramachandran and colleagues [27] described similar sentiments, suggesting that even when practitioners were trained to ask, there were no policies to guide them in what to do next:

*We’re trained to ask the questions, we’re trained to make sure, are you feeling safe, blah, blah, blah. But then someone says ‘yes’ and then you’re like, oh no, because now I really have no idea what to do with them… I’ve never had any real sense of, OK, now what’s the appropriate follow-up? And obviously, I know that you need it, but do I tell them they can call a hotline? Are they really going to do that? Do I make them an appointment while I’m in the office with them to speak with someone? It’s really hard to know, what we do now…* [27]*. (Sexual health nurse, USA)*

Data from five studies suggested that a further barrier to addressing IPA in health settings was a lack of collaboration amongst the different professions and no sense of working together as a team to address IPA. Many HCPs stated that they thought the responsibility for identifying and responding to IPA ought to sit with a different specialty or profession, either because they felt that the other professions (such as social work) were better equipped or because their own role description actively discouraged screening.

*I think they’re [patients] being screened as they come through the emergency department, so I don’t think that screening them again in the fracture clinic adds anything* [32]*. (Fracture clinic, Canada)*

*Not us, I think the doctor is the one who [is] supposed to refer them to the social workers, because we can’t refer patients as nurses. We don’t refer* [47]*. (Emergency nurse, South Africa)*

*Screening for IPV is not our role as nurses and it is not written in the job description, so I have no authority for IPV screening, and could be fired if taking the responsibility for doing that* [20]*. (Nurse, Jordan)*

Lastly, HCPs across multiple healthcare settings highlighted a structural disconnect between healthcare settings and social agencies that support people affected by IPA. HCPs did not feel confident in knowing what referral options were available and how services could help:

*…What would I do if all these people disclosed abuse? Where would I send them for help? Such things can’t work without appropriate mechanisms within the health care system* [52]*. (Primary care physician, Greece)*

*Unfortunately, the referral system is terrible, so I didn't know where to refer her to* [47]*. (Emergency nurse, South Africa)*

In extreme cases, not knowing where to refer individuals encountering IPA for support and feeling cut off from the service sector meant that some HCPs felt it was ‘better not to know’ about IPA. As a family physician in an American study commented:

*If you don’t have the resources... sometimes it makes you reluctant to screen for it. Sometimes you’d rather not know. I mean now all of a sudden they’ve got this woman who is being abused and you can’t do anything and you don’t have the resources to be able to offer her care...that may be a barrier* [26]*. (Family physician, USA)*

#### Societal attitudes enable blaming women

The final theme, reflected across 20 of 43 studies [22, 24–26, 29, 30, 37, 38, 40–42, 44, 47, 53, 54, 57–60, 62], suggests that, in part, the low priority given to the issue of IPA within healthcare settings stems from problematic attitudes and beliefs in wider society that put the reason for not asking or disclosing onto the victim. This includes a perception that women will not disclose due to normalisation of IPA, that IPA only presents in certain types of women, that women will lie or are not reliable patients.

One example of this is the perception that women do not want to disclose IPA to a HCP and are likely to deny it if asked, which was mentioned in seven studies. In low-and-middle-income countries, this perceived reluctance to disclose was linked to patriarchal gender roles and the normalisation of violence. For example, a nurse in a Jordanian study [20] explained that:

*In our culture, women are expected to not disclose IPA, and will not tell the truth. They will tolerate and accept violence for the sake of their own and family dignity and reputation* [62]*. (Nurse, Jordan)*

However, the perception that it is pointless to ask women about IPA was also held by HCPs.

*They are afraid they will not be able to escape, that the situation cannot be resolved, that nothing can be done. No one can help, they are powerless and trapped in it. These people probably do not have an alternative: if they could, they would probably put things in order and leave* [58]*.(Doctor, Slovenia)*

A further perceived barrier to addressing IPA are societal assumptions regarding the types of people affected. HCPs described the belief that IPA is something that happens to ‘other’ people, not their patient cohort:

*Domestic violence is not that common in the group of patients I see because I usually see girls from good, educated, well off families...but in lower classes, less educated, less resources, yes I would say there it is a problem* [37]*. (Doctor, Pakistan)*

*Well, you can find violence in all parts of society, but I do not feel that our women are among the most deprived people. Thus, it’s not … These are not people who have a lot of issues, neither economic nor other problems* [57]*. (Midwife, Norway)*

*You have people who. . . you know very well, you know who their partners are, you see them in the practice. . . it may not even occur to you that person could be violent, so that’s probably why you may not [ask]—I may not so much for somebody I know well* [38]*. (General practitioner, UK)*

A further societal belief that acts as a barrier to IPA identification is that women fabricate or provoke violence. Some HCPs suggested that women are not reliable patients; in particular, those who are intoxicated or mentally ill were highlighted as patients difficult to believe. Additionally, it was suggested that women use allegations of IPA for attention-seeking behaviour:

*While I understand that there are lots of people out there who are abused and we need to screen them and get them help if they want it, at the same time, when you ask the same questions to everyone, sometimes it just offers an invitation for more attention-seeking behaviors* [24]*. (Emergency department nurse, USA).*

## Discussion

This qualitative meta-synthesis updated previous reviews by Sprague [6] and Saletti-Cuesta [7], exploring the perceived personal and structural barriers for health practitioners to identify IPA. We chose to focus on structural barriers in this review as we have published a recent review of personal barriers to addressing IPA [8]. These personal barriers included HCPs not wanting to interfere, feeling like they don’t have control and not wanting to take responsibility for addressing IPA. Whilst a review of the key elements that promote HCP readiness to respond to IPA did identify such personal factors as being important [63], a critical part of being “ready” to respond is having support from the broader healthcare system. Thus, it is likely that the general lack of identification across health settings is also a result of structural issues, including health systems and the societal structures HCPs practice within [4]. Indeed, our findings show that structural barriers exist at the environmental level, the broader health system level and at the societal level.

HCPs in this review felt that the barriers of lack of time and privacy were where the *environment works against us*, consistent with previous reviews [6, 7]. Sprague and colleagues in 2012 emphasised lack of time but not privacy issues, with Sellati-Cuesta and colleagues more recently emphasising privacy and confidentiality as a barrier. Issues about privacy concerns may relate to the healthcare system’s modernisation over time (62) with increasing utilisation of new technology (63). This finding has implications for current practice, given the growth of telehealth use during the COVID-19 pandemic [64]. We acknowledge telehealth use can have potential benefits, for example, calls can be taken outside of the home, away from the perpetrator, providers can be outside of the community to avoid recognition. However, there are also potential harms highlighted by recent Covid-19 lockdowns which prevent HCPs from recognising potential visual signs of abuse. Findings here reinforce the importance of allowing HCP’s the time and privacy necessary to identify IPA and support the needs of patients [63].

At the health system level, HCPs felt they were *tackling the problem on my own*. They felt unprepared by lack of training, and unsupported by colleagues, the organisation, and the health system more broadly. HCPs need to have the support of the team and the health system to be enabled to do this work [63]. This lack of support may reflect the low priority given to the issue of IPA within healthcare curriculums and health service delivery settings [4]. Further, the perception of IPA as a social issue rather than a medical one suggests that appropriate policy, training and cultural reform needs to occur to improve practitioner preparedness to address IPA [63]. Moreover, as long as there remains a deficit in social services, support and limited coordination between HCPs, even if we remove the structural barriers, we will still find deficits in our ability to support IPA patients.

Lastly, we found that some HCPs’ views reflected broader *societal attitudes that enable blaming women* for the lack of identification*.* Problematic attitudes and beliefs include normalisation of IPA and victim blaming which impede IPA identification for some HCPs. It is not surprising that societal beliefs held by some HCPs act as barriers to identifying IPA among their patients. Previous reviews have also touched on how cultural challenges and negative presumptions around IPA-affected women are barriers [6, 7]. Further, women survivors are often seen by parts of the community as unreliable, mentally unwell, and/or apt to not tell the truth [65]. Overall, this finding supports the idea that societal beliefs may influence HCP identification practices, potentially reducing IPA identification opportunities.

### Strengths & limitations

A strength of this meta-synthesis is the diverse range of countries that were represented in the synthesis, as well as the representation of over ten different types of health and allied health professional groups. Several limitations also need to be acknowledged. Firstly, while the CASP [17] is considered a robust method of appraisal and used widely, it is not universally accepted that critical appraisal checklists for qualitative studies are beneficial. Moreover, we could have used the full CERQual [66] to assess the strength of the findings. Our results should thus be interpreted with some caution. Finally, our review excluded non-English language studies that otherwise met the criteria for inclusion, and three studies that our project team was unable to source full-text versions.

## Conclusion & implications

An updated synthesis of the literature was warranted to explore the contemporary evidence on this complex area of structural barriers to identifying IPA. A separate meta synthesis [67] on what advice survivors give on ways to improve disclosure mirrors our findings. This advice includes making the environment safe, private and confidential, ensuring survivors are aware of resources, and that non-judgemental supportive attitudes from HCPs are key. We recommend implementation of a health system model [4] for IPA to overcome the structural barriers for HCPs found by this synthesis to enable identification of IPA [80]. This would include improving HCP curriculum, working environments and workflow processes, developing and implementing clear policies and protocols for how to proceed after IPA is identified. Moreover, introducing clinical champions (advanced practitioners) for support of other staff, delineating pathways to resources and referrals and ensuring sufficient social services/victim services infrastructure outside of healthcare settings. While existing health systems are difficult to change our findings may influence future health system design by promoting models to support change at the organisational, practitioner and patient level. Finally, supporting a cultural shift away from negative attitudes towards IPA survivors and promoting social change [68] may result in similar changes in health care workplaces. Future research could explore variations of approaches, barriers, health system types and service delivery between different countries.

## Supplementary Information

**Additional file 1.**

## Data Availability

Not applicable.

## Footnotes

1 This method is a replication of a companion review already published: Tarzia L, Cameron J, Watson J, Fiolet R, Baloch S, Robertson R, Kyei-Onanjiri M, McKibbin G, Hegarty K: Personal barriers to addressing intimate partner abuse: a qualitative meta-synthesis of healthcare practitioners’ experiences. *BMC Health Services Research* 2021, 21(567).
