# Supplementary material for: Health practitioners’ perceptions of structural barriers to the identification of intimate partner abuse: a qualitative meta-synthesis
Source: BMC Health Serv Res. 2022 Jan 22;22:96. doi: 10.1186/s12913-022-07491-8 (PMC8783157; doi:10.1186/s12913-022-07491-8)
Supplement: Supplementary file 1 — Additional file 1. [file 12913_2022_7491_MOESM1_ESM.docx]

| **Qual meta-synthesis Data Extraction** | **Health practitioner's perceptions of barriers to addressing IPV** | | | |
| --- | --- | --- | --- | --- |
| **Reviewer** |  | | | |
| **Date** |  | | | |
| **1.** | **Identification** | | | |
| **Ref ID (extraction tracker)** |  | | | |
| **Authors** |  | | | |
| **Year** |  | | | |
| **Title** |  | | | |
| **2.** | **Eligibility** | | | |
| **Is the study eligible for inclusion? (Group 1 or 2)** |  | | | |
| **Reason for exclusion** |  | | | |
| **3.** | **Settings and demographics** | | | |
| **Location of study (city, district, state, country)** |  | | | |
| **Language** |  | | | |
| **# of participants (total)** |  | | | |
| **Participant type (e.g. rural/urban, adolescents/older)**  **(Settings and role).** |  | | | |
| **Participants’ demographic characteristics**  **(Age, gender, race, length of experience etc.).** |  | | | |
| **4.** | **Study design** | | | |
| **Aim or objectives** |  | | | |
| **Study design** |  | | | |
| **Study duration (if known).** |  | | | |
| **How was the sample formed or recruited?** |  | | | |
| **Concerns about bias in the study methodology (indicate if own concerns or author's concerns). Limitations of the study** |  | | | |
| **How many sites included in the study?** |  | | | |
| **What data collection method was used? (i.e.: focus group, in-depth interview, survey).** |  | | | |
| **Description of data collection.** |  | | | |
| **5.** | **Ethics** | | | |
| **How were ethical issues addressed?** |  | | | |
| **6.** | **Data analysis** | | | |
| **What data analysis method was used (i.e.: grounded theory, thematic analysis, description of qualitative analysis).**  **Make a note of any theory used too.** |  | | | |
| **7.** | |  | | |
| **Theme (author's interpretation)** | | **Author's interpretation (author text include page numbers)** | **Participant quotation (include page numbers)** | **Reviewer comments** |
|  | |  |  |  |
|  | |  |  |  |
|  | |  |  |  |
| **8.** | | **Author’s conclusions** | | |
|  | | | | |

OTHER COMMENTS:
